# Toscana virus non-structural protein NSs acts as E3 ubiquitin ligase promoting RIG-I degradation

**DOI:** 10.1371/journal.ppat.1008186

**Published:** 2019-12-09

**Authors:** Gianni Gori Savellini, Gabriele Anichini, Claudia Gandolfo, Shibily Prathyumnan, Maria Grazia Cusi

**Affiliations:** Department of Medical Biotechnologies, University of Siena, Siena, Italy; University of Pennsylvania Perelman School of Medicine, UNITED STATES

## Abstract

It is known that the non-structural protein (NSs) of Toscana virus (TOSV), an emergent sandfly-borne virus causing meningitis or more severe central nervous system injuries in humans, exerts its function triggering RIG-I for degradation in a proteasome-dependent manner, thus breaking off the IFN-β production. The non-structural protein of different members of Bunyavirales has recently appeared as a fundamental protagonist in immunity evasion through ubiquitination-mediated protein degradation targets. We showed that TOSV NSs has an E3 ubiquitin ligase activity, mapping at the carboxy-terminal domain and also involving the amino-terminal of the protein. Indeed, neither the amino- (NSsΔN) nor the carboxy- (NSsΔC) terminal-deleted mutants of TOSV NSs were able to cause ubiquitin-mediated proteasome degradation of RIG-I. Moreover, the addition of the C-terminus of TOSV NSs to the homologous protein of the Sandfly Fever Naples Virus, belonging to the same genus and unable to inhibit IFN-β activity, conferred new properties to this protein, favoring RIG-I ubiquitination and its degradation. NSs lost its antagonistic activity to IFN when one of the terminal residues was missing. Therefore, we showed that NSs could behave as an atypical RING between RING (RBR) E3 ubiquitin ligases. This is the first report which identified the E3 ubiquitin ligase activity in a viral protein among negative strand RNA viruses.

## Author summary

Toscana virus is an emergent sandfly-borne virus mainly transmitted to humans by phlebotomine sandflies, which can cause meningitis or more severe central nervous system injuries in some subjects. As many other RNA viruses, it counteracts IFN-β expression by its non-structural protein. Our results expanded our knowledge about the molecular mechanisms by which TOSV exerts its activity as an E3 ubiquitin ligase. This is the first example of a viral protein presenting this activity among negative-strand RNA viruses. Thus, the recognition of this activity and its substrates among viruses are of primary importance to understand how viruses can alter their fitness by the ubiquitin pathway and provide an attractive target for the development of antiviral therapies.

## Introduction

Toscana virus (TOSV; *Phenuiviridae* family, Phlebovirus genus) is an emergent sandfly-borne virus mainly transmitted to humans by phlebotomine sandflies [1–3]. A large number of infections is asymptomatic, however, TOSV infection is the leading cause of meningitis or more severe central nervous system (CNS) injuries, such as encephalitis and ischemia during the summer season in southern Europe [4–5]. The viral genome is composed of the large (L), medium (M) and small (S) segments [6]. The L segment encodes an RNA-dependent RNA-polymerase, the M segment encodes the envelope glycoproteins (Gn and Gc) and a non-structural protein (NSm) and the S segment encodes a nucleocapsid (N) protein and a non-structural (NSs) protein [6, 7, 8, 9]. The NSs protein of some members of the *Phenuiviridae* family represents an important virulence factor being a potent antagonist of type I interferons (IFN-α/β), the main protagonists of the host innate immunity against viral infections. The signaling pathway leading to the secretion of IFN-β and the establishment of an antiviral state are achieved by the induction of the cytoplasmic viral sensors RIG-I (Retinoic-acid Inducible Gene I) and MDA-5 (Melanoma Differentiation Associated gene-5) able to recognize dsRNA molecules generated during viral replication [10]. In order to overcome this first-line defense implemented by the host, viruses have evolved protein(s) able to block IFN-β production and its downstream activity at different steps in the signaling cascade. Among Bunyavirales, Toscana virus (TOSV), the Bunyamwera Virus (BUNV), La Crosse Virus (LACV), Sin Nombre (SNV), Tula (TULV) and Puumala (PUUV) Hantaviruses, Rift Valley Fever Virus (RVFV) and Severe Fever with Thrombocytopenia Syndrome Virus (SFTSV), express the NSs protein acting as a suppressor of IFNs [11–21]. Along with this evidence, previous studies have shown that TOSV NSs could exert its function by triggering RIG-I for degradation in a proteasome-dependent manner, thus breaking off the IFN-β production and blocking the establishment of an efficient antiviral state [21, 22]. The proteasomal degradation of proteins by ubiquitination is a process consisting of a covalent attachment of ubiquitin to target proteins. The molecular machinery which leads to the assembly and linkage of poly-Ub chains to the target protein consists of three enzymes defined as ubiquitin-activating enzymes (E1), ubiquitin-conjugating enzymes (E2) and ubiquitin-ligases (E3), which work sequentially in a cascade. In this context, the E3 ubiquitin ligase is the only enzyme which confers specificity to this system by recognizing a selected target protein [23, 24, 25]. E3 ligases are distinguished in RING (Really Interesting New Gene), HECT (Homologous to the E6-AP Carboxy Terminus) and RBR (RING Between RING). A notable distinction in the mechanism among the classes is that RING E3 catalyzes a direct transfer of ubiquitin from E2 to the target protein, whereas a transfer of ubiquitin by HECT E3 involves an intermediate step where the ubiquitin is first transferred from E2 to an active cysteine residue on HECT E3 ligase, then it is conjugated to the target protein [26–35]. RBR combines properties of RING and HECT E3s to conjugate Ub to target proteins. [32]. Based on the linkage generated between ubiquitin moieties, the cognate proteins undergo regulation of their physiological functions, although the role of some chains is still elusive [36–41]. The non-structural protein of different members of Bunyavirales has recently appeared as a fundamental protagonist in virus replication and immunity evasion through ubiquitination-mediated protein degradation [22, 42–45]. To better understand the interaction between the ubiquitin system and TOSV NSs, we investigated whether this viral protein could be responsible for ubiquitin modifications of specific targets. In this study, we showed that TOSV NSs has an E3 ubiquitin ligase activity mapping at the carboxy- and the amino-terminal domains of the protein. Indeed, it appears to promote the transfer of ubiquitin to RIG-I, thus favoring its proteasome-dependent proteolysis. During the course of evolution and adaptation, many of the large DNA viruses have shown to encode their own Ub modifying machinery to facilitate viral replication through regulating immune responses. Here, we present the first report which identifies the E3 ubiquitin ligase activity in a viral protein among negative-strand RNA viruses, to mediate host–virus interactions.

## Results

### NSs and RIG-I expression in Toscana virus infected cells

Previous results have shown that TOSV was able to induce a RIG-I-mediated IFN-β expression in infected cells, likely because NSs was expressed at a low level and relatively late during the viral replication cycle [18]. Therefore, only the *in vitro* over-expression of NSs could evidence its properties mediating a decrease of RIG-I, due to the ubiquitination and proteasomal degradation of RIG-I after their interaction [21]. To better characterize the role of NSs during TOSV infection, the IFN-β competent cell line, Lenti-X 293T, was infected and analysed. The immunoblotting evaluating the expression of endogenous RIG-I performed on cell lysates of mock-infected, TOSV infected, or poly(I:C) stimulated cells showed that RIG-I was induced as revealed at 24h and 48h post-infection (p.i.), alongside with the NSs protein. To better address whether the lack of endogenous RIG-I degradation in TOSV infected cells was due to the low amount of the non structural protein in the early phase of replication, an over-expression of NSs was performed by transient transfection in infected cells. The immunoblotting of these cell lysates confirmed that the level of endogenous RIG-I was reduced in the presence of a higher amount of NSs (S1 Fig, S1 Dataset). Therefore, as previously reported [18], we might hypothesize that the fast replication of TOSV was able to trigger an early innate immune response by inducing IFNs, and that the NSs protein could not counteract this effect, as it was produced later during the virus replication cycle. (S1 Fig, S1 Dataset). As expected, stimulation with poly(I:C) strongly induced cellular accumulation of RIG-I in the selected cell line and the over-expression of TOSV NSs was able to contrast poly(I:C) effects (S1 Fig, S1 Dataset).

### NSs contains an E3 ubiquitin ligase activity *in vitro*

**Fig 1:**
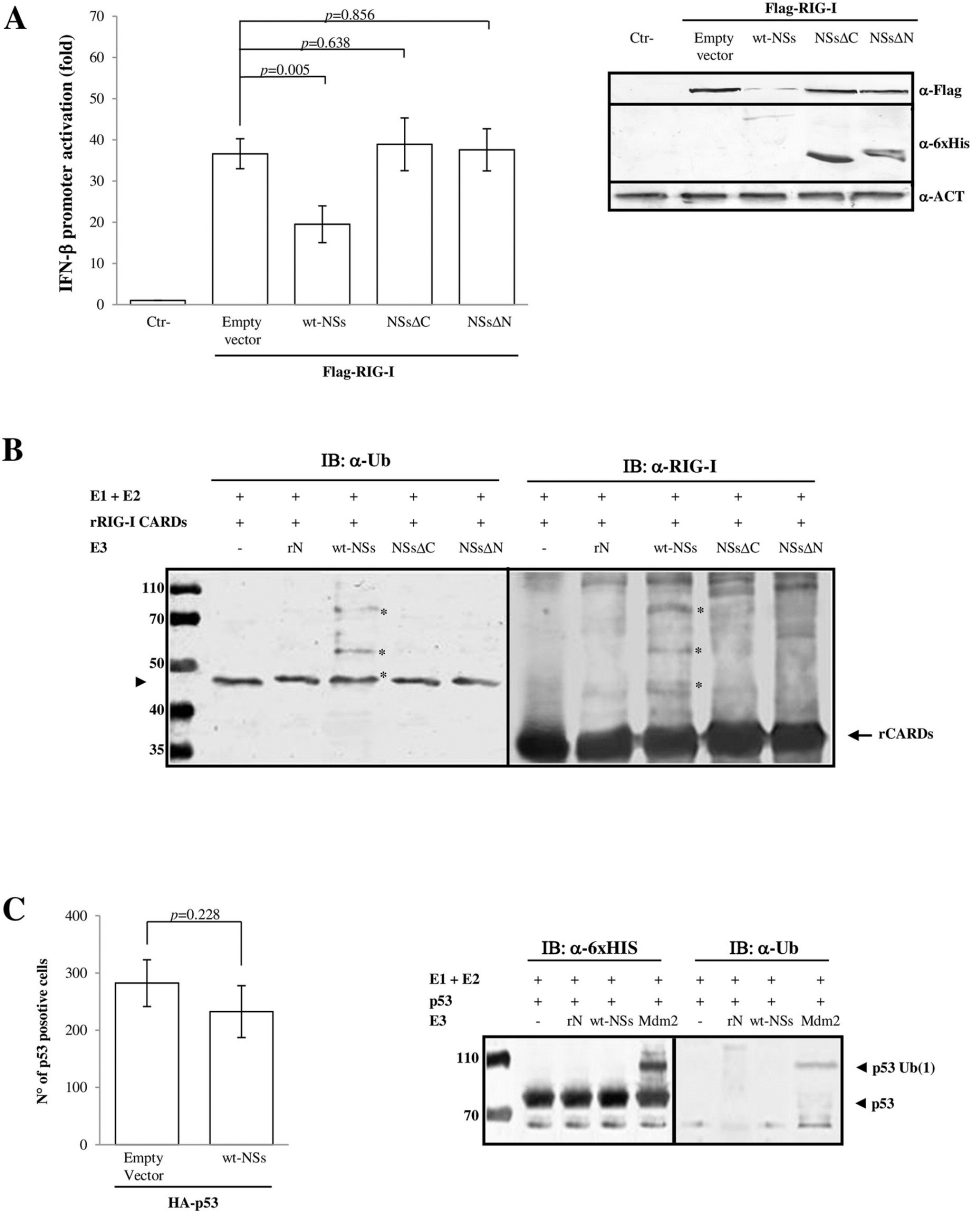
NSs acts as E3 ubiquitin ligase on RIG-I CARDs. Toscana virus NSs inhibits the IFN-β promoter activation through the RIG-I signalling pathway mediating its degradation. Lenti-X 293T cells were transfected (A) with IFN-β promoter-driven FireFly Luciferase (p125-FFLuc) reporter plasmid, expression plasmid encoding FLAG-RIG-I and wild-type (wt-) NSs, as well as deleted NSs expression plasmids, as indicated. In addition, pSV40-RenLuc plasmid was added as internal control. Luciferase activity was analyzed at 48h post-transfection by the Dual-Luciferase Reporter assay as described by the manufacturer (Promega). Relative luciferase activities were measured as fold induction (relative to the basal level of reporter genes in the presence of empty vector after normalization with co-transfected RenLuc activities). Values represent means of triplicate independent experiments ± standard deviations (SD). Representative western blot showing the protein expression levels in the reporter gene assay samples was done on whole cell extracts, resolved by sodium dodecyl sulfate(SDS)-polyacrylamide gel electrophoresis (PAGE) and analyzed by immunoblotting with the FLAG-RIG-I, 6xHis-NSs and β-Actin specific antibodies and densitometric analysis (Supplement data 2). (B) Recombinant proteins were used in the *in vitro* ubiquitination assay using UbcH5b/c as E2 and wt-rNSs, rNSsΔC or rNSsΔN as source of E3 ubiquitin ligase. The negative controls were represented by rRIG-I CARDs tested with the ubiquitination reagents except for E3 Ub ligase or TOSV nucleoprotein (rN) in place of E3 Ub ligase. The ubiquitinated rRIG-I CARDs were detected with anti-ubiquitin (left panel) or anti-RIG-I (right panel) antibodies. The ubiquitinated forms of rCARDS (indicated by asterisk) are present only in the samples containing wt-rNSs, as demonstrated by mass-spectrometry (S4 Fig). The band indicated by arrowhead (left panel) corresponding to the ubiquitinated-E2 (Ub-E2) intermediate present in all the tested samples, comigrates with the monoubiquitinated-rRIG-I CARDs in presence of rNSs. (C) NSs does not affect p53 expression in cells transfected with HA-p53 and 6xHis-NSs expressing plasmids (left panel). Immunofluorescence was performed with indicated specific antibodies; positive cells were counted. The bars depict the average number of p53 positive cells in the presence or absence of wt-NSs. The mean ± SD of three independent experiments is shown. The specificity of the E3 ubiquitin ligase activity of wt-NSs was evaluated in the *in vitro* ubiquitination assay using UbcH5b/c as E2 and recombinant p53 protein as acceptor target for ubiquitination (right panel). Poly-ubiquitinated forms were detected by anti-6xHis tag and anti-ubiquitin antibodies. Higher molecular weight bands corresponding to ubiquitinated p53 were detected only in the reaction supplemented with Mdm2 E3 ubiquitin ligase, but not in the samples containing wt-rNSs or rN.

Previous results [18, 21, 22] have shown that TOSV NSs presented inhibitory properties versus the IFN-β mediated immune response. In particular, RIG-I was targeted for proteasomal-degradation through the action of NSs, and its functional activity was related to the carboxyl-terminus of the protein itself [22], however, assuming that other domains could also be involved. Therefore, we also tested the activity of the amino-terminus (71 aa.) deleted NSs protein (NSsΔN) (S2 Fig) towards RIG-I. Surprisingly, we found a behavior similar to the one observed for the carboxy-terminus deleted NSs (NSsΔC). NSsΔN significantly lost its degrading activity on RIG-I upon co-transfection of cells with the respective plasmids (Fig 1A, S2 Dataset). Moreover, RIG-I-mediated IFN-β promoter activation was not affected by NSsΔN, as shown by the luciferase reporter assay (*p* = 0.638) (Fig 1A, S2 Dataset). Since the treatment with the proteasome inhibitor MG-132 reversed RIG-I degradation by NSs [21], the possibility of a ubiquitin-mediated proteasomal degradation was evaluated. A growing number of viruses was found to weaponize the ubiquitin modification system to suppress IFN [11–21]. Thus, looking for a tool to identify a functional site of the protein, we submitted the NSs protein sequence to the Phyre2 prediction software (www.sbg.bio.ic.ac.uk/phyre2/html/page.cgi) [46], which provided an alignment of 15 residues (aa. 277–292) and a confidence of 30.7% between NSs and RNF31, the E3 ubiquitin-protein ligase. Considering the potentiality of NSs both to act as an E3 ligase and its ability to induce proteasome degradation of RIG-I, we investigated whether TOSV NSs could have this activity *in vitro*. The His-tagged recombinant NSs protein was produced in bacteria, purified and tested for the E3 ubiquitin ligase activity, as described in Materials and Methods. In such reactions, the ability of a protein to promote a ubiquitin-protein ligation was indicative of an E3 ligase activity. For this purpose, an *in vitro* biochemical assay was performed with E1, E2, rNSs and recombinant full-length RIG-I (FL-rRIG-I). When the wt-NSs was used as a source of E3 Ub ligase in the assay, a shift in RIG-I molecular weight, corresponding to its ubiquitinated form, was revealed by immunoblotting using anti-Ub or anti-RIG-I antibodies (S3 Fig). In subsequent experiments, the recombinant N-terminus of RIG-I containing two tandem-repeated CAspase Recruitment Domains (CARDs), necessary and sufficient to activate RIG-I and induce the recruitment of downstream signaling molecules, were tested. Likewise, when the E3 Ub ligase was substituted by the wt-NSs in the assay, higher molecular weight ubiquitinated bands were revealed by immunoblotting on rRIG-I CARDs, using anti-Ub or anti-RIG-I antibodies (Fig 1B). In order to better evaluate which domain of NSs could have a role in the ubiquitination process, the His-tagged recombinant NSs protein, NSsΔC and NSsΔN deleted proteins were produced in bacteria, purified and tested for the E3 ubiquitin ligase activity. Ubiquitinated rRIG-I CARDs bands were not present when NSsΔC, NSsΔN or TOSV nucleocapsid (N) proteins were added in place of NSs (Fig 1B), indicating that NSs possessed an E3 Ub ligase activity directed to RIG-I. Furthermore, as this activity was not observed in deletion mutants, it was likely located in the amino- and carboxy-terminus regions. In the attempt to investigate the potential substrate specificity of TOSV NSs protein, we tested NSs with the recombinant human p53 protein, known to be ubiquitinated by many different E3 ligases. The co-expression of NSs and p53 plasmids in cells did not reveal any decrease of the p53 protein, in comparison with the control represented by cells transfected with p53 plasmid alone, as evidenced by immunoblotting and immunofluorescence (Fig 1C). Moreover, in biochemical assays, p53 was ubiquitinated by Mdm2 E3 Ub ligase, and not by NSs (Fig 1C), indicating that its activity appeared to be target specific. Finally, ubiquitinated products were not detected in the negative controls, when E3 ubiquitin ligase was omitted or substituted by TOSV N protein in the reaction (Fig 1C).

### E3 ubiquitin ligase activity is associated with the C-terminus of NSs

**Fig 2:**
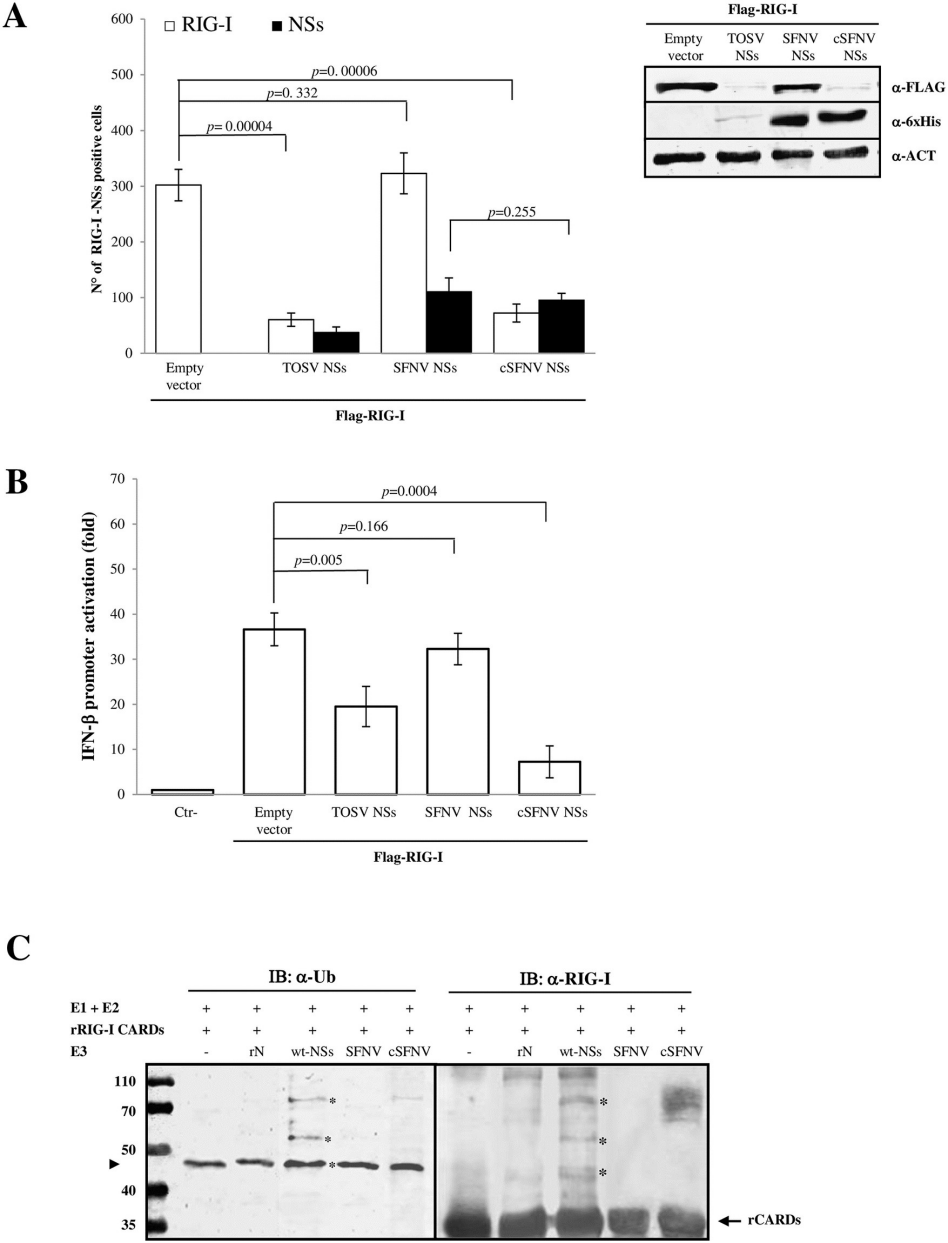
C-terminus of TOSV NSs is linked to E3 ubiquitin ligase activity. The fusion of TOSV NSs C-terminus to Sandfly Fever Naples Virus (SFNV) NSs conferred it a different behaviour. (A, left panel) The chimeric protein cSFNV NSs was tested for its degrading activity on RIG-I co-transfected cells, by immunofluorescence, using specific antibodies [RIG-I (□), NSs (■)]. Graphs are based on the mean values of three independent experiments ± SD. (A, right panel) A more accurate analysis of cellular RIG-I degradation was performed in co-transfected cells by immunoblotting on the whole cell lysates using anti-FLAG or anti-6xHis antibodies. The intensity of the RIG-I band was quantified by densitometry (Supplement data 3). (B) Lenti-X 293T cells were transfected with IFN-β reporter plasmid, FLAG-RIG-I expression plasmid along with TOSV wt-NSs, SFNV wt-NSs or chimeric cSFNV NSs expressing plasmids. Luciferase activities were measured after poly(I:C) treatment. Fold induction was calculated for each sample with respect to the basal empty plasmid transfected sample, after normalization of the signal with the pSV40-RenLuc internal control. The mean values of at least three sets of experiments ± SD are presented. C) cSFNV NSs showed E3 ubiquitin ligase activity in the biochemical reaction, in association with UbcH5b/c as E2. Poly-ubiquitinated bands of rRIG-I CARDs were detected by immunoblotting using anti-RIG-I or anti-Ub antibodies when cSFNV or TOSV NSs was used in the reaction in place of E3 Ub ligase. No ubiquitination activity was shown when SFNV NSs was used.

In order to confirm whether NSs E3 ubiquitin ligase activity, required for targeting RIG-I to proteasomal degradation, mapped to the C-terminal sequence of the NSs, we adopted a ‘twist’ strategy and constructed a TOSV-Sandfly Fever Naples Virus (SFNV) NSs chimeric protein. Toscana virus and Sandfly Fever Naples virus (SFNV) belong to the same viral genus and share a high sequence homology in the non-structural protein (54%) (S4 Fig). However, SFNV NSs lacks the last 78 aa, present in TOSV NSs. Since this domain was proved to be strikingly associated to TOSV NSs degrading activity on RIG-I [22], we generated a chimeric SFNV NSs (cSFNV) protein by fusing the C-terminus of TOSV NSs onto the NSs of the related SFNV. In comparison with TOSV NSs, SFNV NSs was well expressed in transfected cells and did not show any RIG-I-mediated IFN-β inhibition, since unable to mediate RIG-I degradation (*p* = 0.166) (Fig 2A and 2B). Thus, we supposed that the addition of TOSV C-terminus to SFNV NSs could confer new properties to this protein. Analysing the cSFNV NSs expression by immunofluorescence and immunoblotting, no significant difference was evidenced with the wild-type counterpart (*p* = 0.255) (Fig 2A). On the other hand, the addition of a partial TOSV sequence completely altered the SFNV NSs function. Indeed, an evident decrease of RIG-I was revealed in cSFNV and FLAG-RIG-I plasmids transfected cells, showing few RIG-I positive cells by immunofluorescence (*p* = 0.00006) and a faint band of RIG-I by immunoblotting (Fig 2A, S3 Dataset). Furthermore, the newly acquired IFN-β antagonistic property of cSFNV NSs was confirmed by luciferase reporter assay, demonstrating a strong inhibition of RIG-I-mediated IFN-β promoter activation (Fig 2B) and endorsing the hypothesis of an E3 ubiquitin ligase activity related to the C-terminal domain of TOSV NSs. This data was also supported by the *in vitro* ubiquitination assay of rRIG-I CARDs in the presence of the recombinant cSFNV NSs protein. As shown in Fig 2C, rRIG-I CARDs were ubiquitinated when the chimeric protein was added in the reaction, in place of the E3 Ub ligase. On the contrary, SFNV NSs protein did not have any effect on rRIG-I CARDs (Fig 2C). Moreover, the mass spectrometry, performed on the products of the *in vitro* ubiquitination reactions, identified RIG-I CARDs ubiquitinated at the lysine residues 115 and 172 (S5 Fig). Since Ub-CARDs specific peptides were only identified in the wt-NSs and cSFNV containing samples, the E3 ubiquitin ligase activity of TOSV NSs and the incisive role of its C-terminal domain were further confirmed.

### Cysteine _27_ at the N-terminus of NSs is also involved in the ubiquitination process

**Fig 3:**
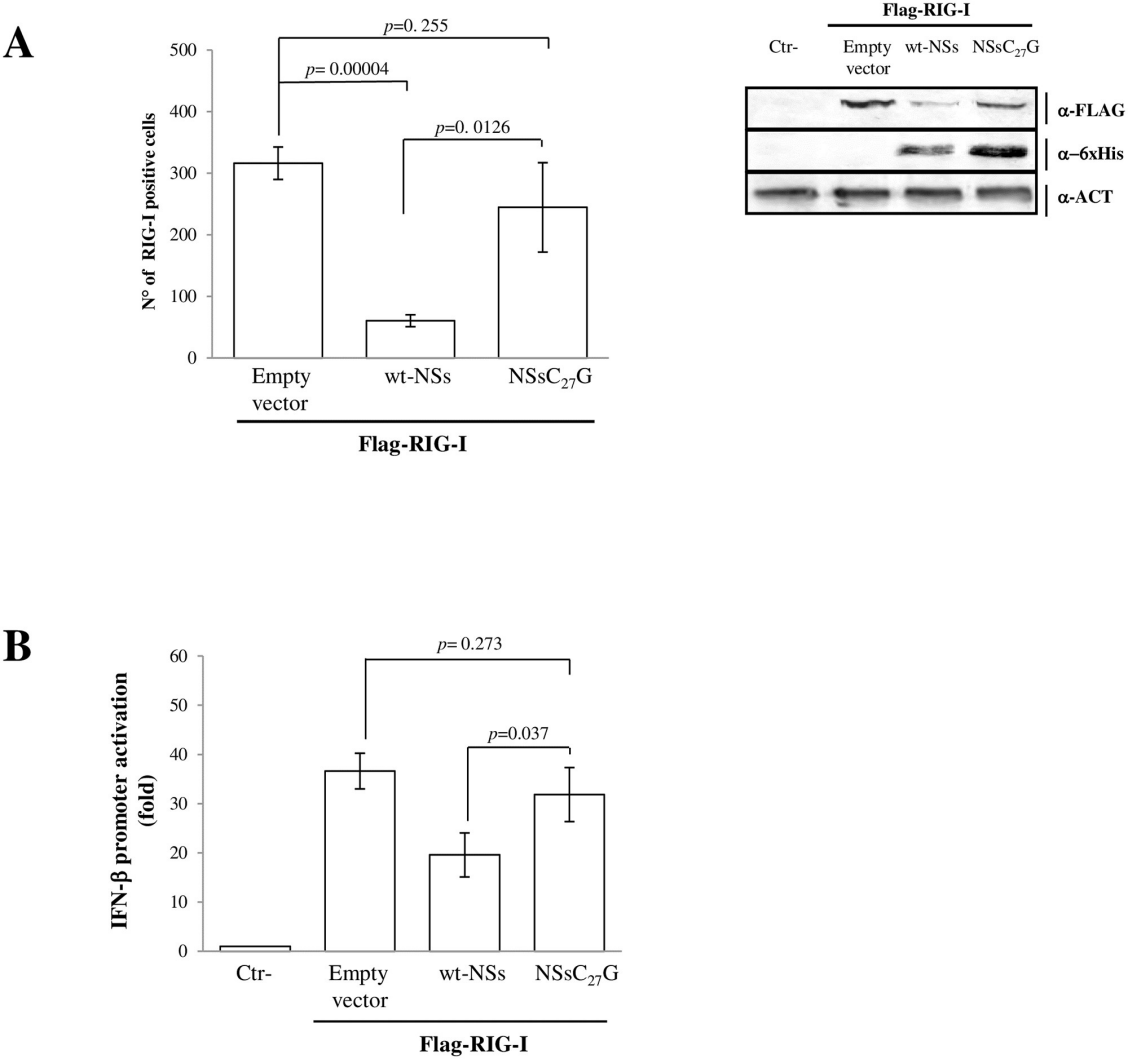
Effects of TOSV NSs N-terminal domain on E3 ubiquitin ligase activity. (A) The involvement of NSs Cystein_27_ in the ubiquitination of RIG-I was evaluated by immunofluorescence on cells co-transfected with RIG-I and NSs plasmids. Cells were stained with anti-FLAG antibody and RIG-I positive cells were counted on different fields. Mean values ± SD of more than three independent experiments were plotted (left panel). Results were validated by immunoblotting (right panel) using total cell lysates of co-transfected cells and semi-quantitative analysis was done by densitometry (supplement data 4). (B) Lenti-X 293T cells were transfected with IFN-β reporter plasmid (p125-FFLuc) along with RIG-I and pSV40-RenLuc plasmids in addition with the wild type-NSs, or C_27_G-NSs mutant or empty plasmids. After stimulation with poly(I:C), luciferase activity was analyzed. For each sample, luciferase was normalized to the RenLuc reporter activity. Data are representative of three independent experiments and are expressed as mean ± SD of normalized luciferase activity.

Since we have demonstrated that NSsΔC and NSsΔN did not have any inhibitory activity to RIG-I mediated IFN-β promoter activation (Fig 1A), we hypothesized that both the C- and N-terminus of NSs might be involved in the ubiquitination of RIG-I. We suspected that NSs could behave such as an RBR E3 Ub ligase, which transfers ubiquitin to a catalytic cysteine on the E3 ligase and then to the substrate. Therefore, a mass spectrometry analysis was performed on the wt-NSs protein, recovered from transfected Lenti-X 293T cells, to see whether a cysteine of NSs was ubiquitinated. The analysis revealed a ubiquitinated cysteine at position 27, in the N-terminal of TOSV protein (S6 Fig). In the recent years, several new modes of ubiquitin chain attachment have emerged and even the thiol groups of cysteine residues could be employed as sites of ubiquitination [47]. However, the potential importance of this ‘non-canonical ubiquitination’ and its roles still need to be elucidated [48]. Therefore, in order to understand the role of this cysteine in the ubiquitination process, we mutated Cys_27_ to Gly in the wt-NSs and tested the new construct in the *in vitro* ubiquitination assay of rRIG-I CARDs. rNSs-C_27_G was unable to ubiquitinate rRIG-I CARDs (S7 Fig) and the result was supported by immunofluorescence, immunoblotting (Fig 3A, S4 Dataset) and luciferase assays (Fig 3B), which revealed a behaviour of rNSs-C_27_G similar to the one observed for NSsΔN and NSsΔC, and indicated the fundamental role elicited by this amino-acid, interfering with cell signaling. Therefore, this result also validated the involvement of the NSs N-terminus in the ubiquitination process.

## Discussion

**Fig 4:**
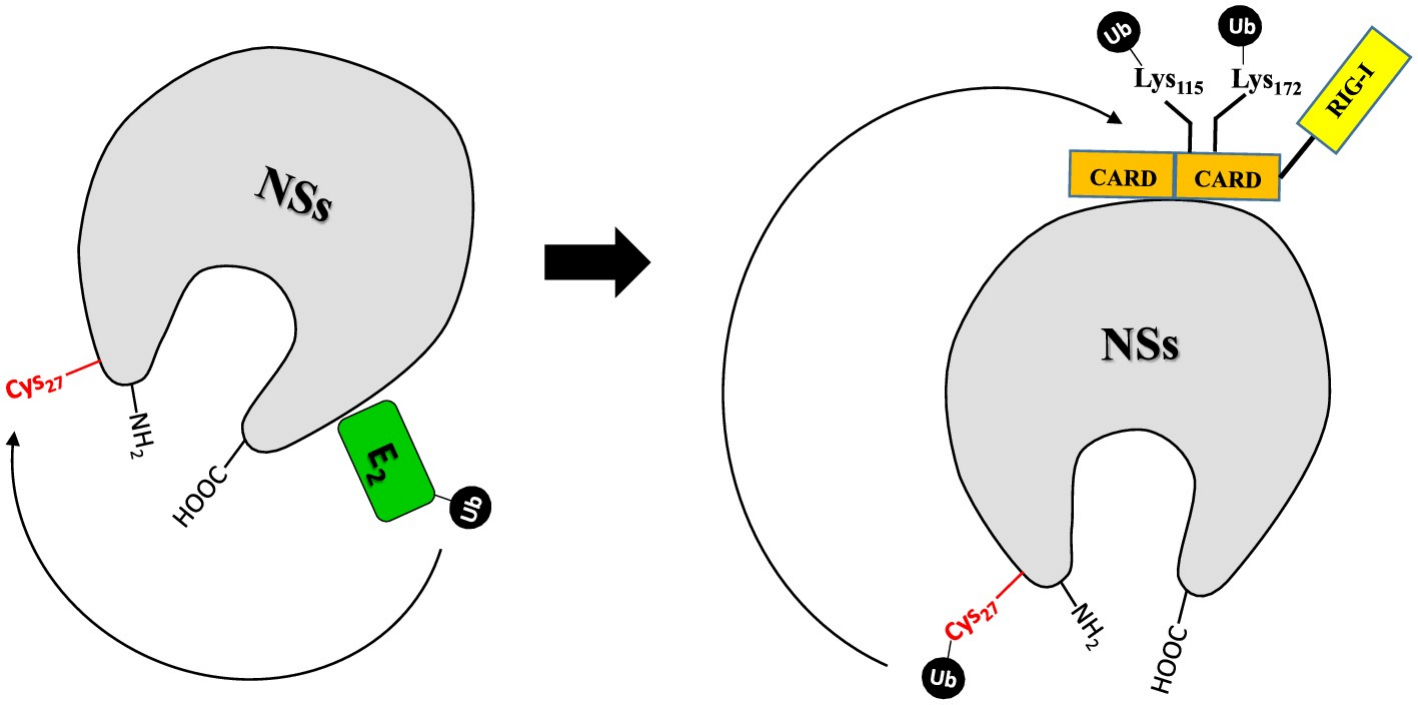
Model for NSs E3 Ub ligase function. Schematic model for TOSV NSs which carries out an unconventional E3 Ub ligase activity (RING between RING; RBR) by a RING-HECT-hybrid mechanism. The model proposes the transfer of ubiquitin from the charged E2 Ub conjugating enzyme, bound to the C-terminal of NSs, to Cysteine _27_ located at the N-terminal of the protein. Then, ubiquitin is transferred to the lysine residues of RIG-I target protein, interacting with the central region of TOSV NSs, and leading to its proteasome-dependent degradation.

Innate immunity is fundamental to protect host cells against pathogens. In turn, viruses have developed different strategies to counteract host innate immune response [11–21, 42–45]. New mechanisms of viral evasion of host immune response, exploiting the ubiquitin system, have recently been described. Indeed, some viruses encode proteins that manipulate the ubiquitin pathway, inhibiting the immune signaling and forwarding the degradation of host proteins [49–53]. Examples are provided by members of the *Herpesviridae* family; Varicella Zoster virus (VZV) encodes ORF61 containing a RING domain, which inhibits the IFN expression, by targeting IRF3 degradation [54]. Similarly, Herpes virus type 1 (HSV-1) has a RING domain in the ICP0 protein that confers E3Ub ligase activity for the degradation of host proteins involved in the innate immunity [55]. Poxviruses behave in a similar way [56]. Thus, ubiquitination has an important role in regulating signal transduction during the immune response [53, 57, 58]. Although viral E3 Ub ligases have been identified in large DNA viruses, some RNA viruses have developed mechanisms to interact with host molecules involved in the ubiquitination pathway. Influenza NS1 protein binds and inactivates the TRIM-25 and Riplet E3 ubiquitin ligase, preventing the downstream activation by K_63_ poly-Ub chain moiety of RIG-I [59, 60, 61]. Likewise, the Paramyxovirus V protein interacts with RIG-I/TRIM-25 regulatory complex by inhibiting RIG-I signaling [62]. Hepatitis C virus NS3-4A protein targets Riplet for degradation [57]; Rotavirus NSP1 triggers the degradation of targets by hijacking a subset of E3 Ub ligases, the cullin-RING ligases [63]. Among *Phenuiviridae* members, Rift Valley fever virus (RVFV) is the most investigated virus for the antagonistic effects of its NSs protein on the innate immune response and recently, the involvement of ubiquitin system, particularly the SCF E3 ubiquitin ligase complex, has been elucidated [44, 58]. Ubiquitin-proteasomal degradation of p62 subunit of the transcription factor TFIIH is a consequence of the interaction between RVFV NSs and the F-box protein FBXO3-SKP1-Cullin1/7 SCF multi-protein complex [43]. Furthermore, RVFV NSs is able to recruit the F-box protein FBXW11 and induce ubiquitination and proteasomal degradation of the antiviral protein PKR [44, 58]. In this study, we have shown how TOSV NSs protein was inducing RIG-I degradation upon their binding [21]. In particular, TOSV NSs revealed an E3 ubiquitin ligase activity related to both the carboxy- and amino-terminal domains of the protein, promoting the transfer of ubiquitin to RIG-I and favoring its proteasome-dependent proteolysis. Indeed, TOSV NSs mutants, such as those deleted at the amino- or carboxy-terminal (NSsΔN, NSsΔC), unlike wt-NSs, were not able to activate IFN induction. This data conferred a new role to the protein terminal sequences and suggested an involvement of these protein regions in the E3 ligase activity. Initially, an E3 ubiquitin ligase activity appeared to be localized at the NSs carboxy-terminal; thus, in order to demonstrate it, the amino-acid stretch 248–316 was fused to the carboxy-terminus of the SFNV NSs, sharing a homology of 54% with TOSV NSs, and lacking this sequence (S4 Fig). SFNV NSs did not show any degrading activity to RIG-I; on the contrary, the obtained chimeric protein acquired the features of TOSV NSs, becoming capable to degrade RIG-I. Then, we demonstrated that neither NSsΔC nor NSsΔN were able to ubiquitinate RIG-I *in vitro*. Thus, it appeared that both the amino-acid ends of TOSV NSs could be involved in an E3 ubiquitin ligase activity. Indeed, NSs, but not NSsΔC or NSsΔN, could transfer ubiquitin to the RIG-I substrate, as shown in the *in vitro* reaction in which ubiquitination process only occurred in the presence of E1, E2 and wt-NSs in place of the E3 Ub ligase. The results were also confirmed by mass spectrometry analysis, which revealed the presence of ubiquitin at positions 115 and 172 of rRIG-I CARDS after the *in vitro* reaction, in the presence of NSs. Moreover, this reaction was specific for RIG-I CARDs, since NSs did not show this activity with a different target, such as the onco-suppressor protein p53. Therefore, it was interesting to understand which type of E3 ubiquitin ligase could be ascribed to TOSV NSs. While some RING or HECT E3 ligases appear to interact directly with the substrate and E2 [25, 26, 29, 31, 64], others require additional components and interact with the substrate only indirectly, as part of a multi-subunit CRL complex [26–35]. Both RING and HECT E3 ligases transfer ubiquitin to a lysine residue on the substrate, RING E3s act as a platform to allow a direct transfer of ubiquitin from the E2 to the substrate [64]. On the other hand, HECT E3s accept ubiquitin from E2 to form a ubiquitin-thioester intermediate with an active cysteine, then transfer ubiquitin to both the ε-amino groups of lysine side chains of the substrate [25, 31]. Thus, cysteine may play a significant role, particularly in the ubiquitin modification for signaling. In our study, the finding of a ubiquitinated cysteine, a non-canonical site [47, 48], at position 27 of TOSV NSs, led us to suppose that this residue could also be involved in the E3 ubiquitin ligase activity, in addition to the C-terminal sequence of the same protein. Indeed, even the C_27_G mutant of NSs was no more able to degrade RIG-I and could induce an IFN promoter in transfected cells, although at a lower level, in comparison to the NSsΔN. Therefore, we hypothesized that NSs could behave as an atypical RBR that has elements of both HECT and RING ligases: one RING domain binds the charged E2, while the other domain accepts the ubiquitin molecule before transferring it onto the substrate [32]. We hypothesized that ubiquitin-conjugated E2, bound to the carboxyl-terminus of NSs, transferred the ubiquitin to Cys_27_ (thioester intermediate), in the amino-terminal, as demonstrated by mass spectrometry, and, from there, to RIG-I, linked to NSs (Fig 4). This model might explain why NSs was losing its antagonistic activity to IFN, when one of the two amino-acid ends was lost. At present, we do not know why cSFNV NSs showed a ubiquitin E3 ligase activity, despite not having a cysteine at position 27, but it is possible that cysteine at position 39 of SNFV NSs was processed in the same way. Further investigations are ongoing in order to better determine the dynamics of the steps in this ubiquitination process; however, this is the first study showing a viral protein with an E3 ubiquitin ligase activity among negative strand RNA viruses. Although viruses have acquired tactics to minimize host antiviral responses by co-evolving with their hosts, RING E3s, encoded or hijacked by viruses for evading immune responses, are largely undiscovered and can clarify how viruses play this game with their host.

## Materials and methods

### Cells and viruses

Vero cells (ATCC CCL-81) and human embryonic kidney Lenti-X 293T cells (Clontech, Milan, Italy) were cultured in Dulbecco’s modified Eagle’s medium (DMEM) (Lonza, Milan, Italy) supplemented with 100 U/mL penicillin/streptomycin (Hyclone Europe, Milan, Italy) and 10% heat-inactivated foetal calf serum (FCS) (Lonza), respectively, at 37 °C. Toscana virus (TOSV) strain 1812 [18] was used for all the experiments described.

### Reagents and antibodies

Transient transfections were performed with GeneJuice Transfection reagent (Novagen, Milan, Italy), according to the manufacturer’s instruction, or standard calcium phosphate method [65]. Chemicals were all purchased from AppliChem GmbH (Germany). The proteasome inhibitor MG-132 was purchased from Sigma-Aldrich (Milan, Italy). Mouse anti-6xHis tag antibody (GE Healthcare, Milan, Italy), anti-RIG-I (DDX58) polyclonal antibody (OriGene, Rockville, MD, USA), mouse anti-FLAG M2 monoclonal antibody (Agilent Technologies, Milan, Italy), mouse monoclonal anti-HA tag antibody, fluorescein (FITC)-labeled anti-mouse IgG and anti-mouse IgG HRP-conjugated were purchased from Sigma-Aldrich. Anti-rabbit IgG HRP-conjugated was supplied by Santa Cruz Biotechnology Inc. (Heidelberg,Germany) Ni-NTA sepharose was purchased from Novagen (Milan, Italy) and anti-Ub FK2 clone from Enzo Life Sciences (New York, USA).

### RIG-I expression in TOSV infected cells

Lenti-X 293T cells were grown in a 24-wells plate (5x10^5^/ml). After 24h, cell monolayers were infected with TOSV 1812, by using a multiplicity of infection (MOI) of 1. After 1h adsorption at 37 °C, viral inoculum was removed and replaced by complete growth medium. Cells were collected at 24h and 48h post-infection (p.i.). Where indicated, infected cells were transfected with 1 μg of NSs expression plasmid or empty plasmid, 5h after infection. Positive control was obtained by stimulating cell with poly(I:C) for 18h. Cell lysates were collected in RIPA buffer; 50 μg of total proteins were resolved by SDS-PAGE and then transferred to nitrocellulose (NC) membrane (Santa Cruz Biotechnology, Heidelberg, Germany). After blocking with 5% non-fat dry milk, filters were incubated O/N at room temperature with anti-RIG-I (1:5000 dilution), anti-NSs (1:200 dilution) or anti-N (1:200 dilution) mouse sera. After being washed with PBS 0.2% Tween-20 (PBS-T), membranes were incubated with anti-mouse HRP-conjugated secondary antibody (1:5000 dilution) and proteins were detected with TMB Enhanced One Component HRP Membrane Substrate (Tebu-bio, Milan, Italy).

### Plasmids

Toscana virus full-length, NSsΔC (nt: 1–861) expressing plasmids were cloned in pcDNA4HisMax (Life Technologies, Milan, Italy) as described elsewhere [22]. Similarly, NSsΔN (nt: 217–537) expressing plasmid was generated by PCR with NSsΔN BamHI sense (nt 217–231) 5’-CGCGGATCCCCATGGCTGTACTGGGGCCT-3’ and NSs EcoRI antisense (nt 948–931) 5’-CCGGAATTCTAAGGGTGGGTAGTGGGG -3’ primers (Sigma-Aldrich). The gene was cloned in pcDNA4HisMax-A plasmid (Invitrogen) at the BamHI-EcoRI unique sites of the polylinker in frame with the 6xHis tag. The Cysteine_27_ mutant was obtained by using QuikChange II Site-Directed Mutagenesis Kit (Agilent Technologies, Milan, Italy), according to the manufacture’s instruction. Sandfly Fever Naples Virus (SFNV), strain Sabin (GenBank Accession N° EF201829) chimeric NSs gene carrying the TOSV C-terminal domain (cSFNV) was generated by PCR using, as reverse primer, a synthetic DNA fragment (gBlock: Integrated DNA Technologies) consisting of the C-terminus of TOSV NSs gene (nt: 739–951) partially overlapping to the 3’-end of SFNV NSs ORF, and a SFNV-NSs sense primer (primers sequences available upon request). The chimeric gene was cloned in the pcDNA4HisMax plasmid (Life Technologies) by standard procedure. Full-length Toscana virus NSs gene was also cloned in the bacterial expression plasmid pET15b (Novagen), while ORFs coding for all the NSs mutants and RIG-I RIG-I CARDs were cloned in pRSET plasmid (Life Technologies). All the recombinant plasmids were confirmed by sequencing. The reporter plasmid encoding Firefly Luciferase downstream the complete interferon-beta promoter (p125-Luc) was kindly provided by Takashi Fujita (Tokyo Metropolitan Institute of Medical Science, Tokyo, Japan) [66], while the *Renilla* Luciferase reporter plasmid (pSV40-RL) was purchased from Promega (Promega, Milan, Italy). Plasmids for FLAG-tagged human RIG-I, RIG-I N-terminal RIG-I CARDs domain (RIG-IN), HA- human p53 and the HA-tagged human ubiquitin were kindly provided by A. García-Sastre (Mount Sinai School of Medicine, New York), T. Fujita (Tokyo Metropolitan Institute of Medical Science, Tokyo, Japan), M. Tommasino (International Agency for Research on Cancer, Lyon, France) and D. Arnoult (Inserm, France), respectively.

### Recombinant proteins expression and purification

Recombinant proteins production was achieved by induction of transformed BL21(DE3)-pLys (Novagen) cells with 1 mM IPTG for 3h at 37 °C. TOSV NSs was recovered from inclusion bodies via solubilisation with 50 mM Tris-HCl [pH 7.5]; 300 mM NaCl; 0.3% w/v N-laurylsarcosine (SRK) and 6xHis tagged fusion proteins were purified by using Ni-NTA sepharose following manufacturer’s instruction. 6xHis-RIG-I CARDs were purified from IPTG induced BL21(DE3)-pLys (Novagen) bacterial culture as described above, with SRK omission. Purified protein fractions were analysed by SDS-PAGE and pure protein containing fractions were pooled, diluted ten-folds with 10 mM Tris-HCl [pH 8.0] and dialysed against the same buffer O/N at room temperature. Recovered proteins were concentrated by using ultrafiltration devices, quantified by BCA reagent (Pierce, Milan, Italy) and stored at -80 °C in aliquots. TOSV recombinant nucleoprotein N was produced and purified as described elsewhere [67]. Recombinant human full-length RIG-I was purchased by BPS Bioscience Inc. (San Diego, CA, USA).

### Immunofluorescence

Lenti-X 293T cells, seeded in 24-wells culture plate, were transfected with 0.5 μg of wt-, deleted- or mutated-NSs expressing plasmids, alone or in combination with 0.05 μg of FLAG-RIG-I or HA-p53 expressing plasmids. Cells were collected and stained with anti-6xHis antibody (1:2000 dilution), anti-HA antibody (1:500 dilution) or with anti-FLAG M2 antibody (1:500 dilution). FITC-labeled anti-mouse IgG (1:320 dilution) was used as secondary antibody. Immunofluorescence was visualized by a Diaplan microscope (Leica Microsystems, Milan, Italy). RIG-I and NSs positive cells were counted in three different fields of the same slide. The mean value of positive cells was calculated with respect to the total number of spotted cells (3.5x10^5^/well).

### Luciferase reporter gene assay

2x10^5^ Lenti-X 293T cells were seeded in 24-well plates and transfected with indicated plasmids as previously described. Briefly, 0.2 μg of p125-FFLuc, 0.05 μg of RIG-I and, where indicated, 0.5 μg of wt-NSs or NSs mutants expressing plasmids were co-transfected. Empty plasmid was used to normalize total DNA amount. Twenty ng of pSV40-RL were co-transfected as internal control. Thirty-six hours post-transfection, cells were stimulated with 2 μg/ml of poly(I:C). After additional 12h, cells were collected and luciferase activities were measured on lysates by using dual Luciferase reporter assay reagent (Promega), according to the manufacturer's instructions. Cell lysates were stored at -20 °C for further analysis by immunoblotting. Results are given as mean values of several experiments ± standard deviations (SD).

### Immunoblot analysis

Fifty μg of total cell lysates of co-transfected cells were resolved by SDS-PAGE and then transferred to nitrocellulose membrane. Immunoblotting was performed as described above, by using anti-HA (1:1000 dilution), anti-FLAG (1:2000 dilution) or anti-6xHis (1:1000 dilution). Quantitative comparison among samples was performed by densitometric analysis using the ImageJ software as reported in Supplement data.

### *In vitro* ubiquitination assay

To evaluate TOSV NSs E3 ubiquitin ligase activity, purified recombinant wt-NSs or its mutants were used in the *in vitro* protein Ubiquitination assay (Enzo Life Science). Experimental reactions and controls were added as suggested by the manufacturer. Briefly, 100 nM ubiquitin activating enzyme (E1); 2.5 μM of ubiquitin conjugating enzyme (E2) UbcH5b/c; 1 μM of rNSs protein or its mutants as source of E3 ligase, 2.5 μM of biotinylated ubiquitin and 1 μM of recombinant purified RIG-I, RIG-I CARDs or p53 were incubated at 37 °C for 4-6h. Negative control reactions lacking rNSs or containing 1 μM of TOSV rN in place of E3 ubiquitin ligase were included in the experiment set. Reactions were quenched by adding 5X gel loading buffer and analysed by western blotting for ubiquitinated RIG-I CARDs by anti-DDX58 polyclonal antibody (1:500 dilution) or anti-Ub FK2 clone (1:1000 dilution).

### Mass spectrometry detection of ubiquitinated protein residues

Lenti-X 293T seeded in T25 flasks were transfected with 3 μg of wt-NSs plasmid in combination with 1 μg of plasmid encoding for HA-tagged ubiquitin. At 36h post-transfection, cells were treated with MG-132 to a final concentration of 1 μM for additional 12h and collected at 48 h post-transfection. Pull-down for NSs was achieved by Immobilized Metal Affinity Chromatography (IMAC) under denaturing conditions. Briefly, cell pellets were lysed in 5 M guanidine-HCl; 10 mM HEPES [pH 8.0] with sonication. His-tagged NSs was bound to Ni-NTA sepharose for 3h at room-temperature. Beads were collected and extensively washed with 10 mM HEPES [pH 8.0], 1 M NaCl, 0.3% w/v SRK, 50 mM imidazole. Bound proteins were eluted with Laemmli denaturing sample buffer, loaded on SDS-PAGE and stained with Bio-safe Coomassie stain (Bio-Rad, Milan, Italy). Protein bands were cut from gel and prepared for mass-spectrometry analysis as carried out by Cogentech Proteomics/MS (Cogentech S.c.a.r.l., Milan, Italy), by using the nLC-ESI-MS/MS QExactive-HF system. Similarly, the rRIG-I CARDs ubiquitination was confirmed by mass-spectrometry performed on the *in vitro* ubiquitination reaction products.

### Statistical analysis

The mean differences were statistically analyzed using Stat View statistical software (Abacus Concepts, Berkeley, CA). Immunofluorescence and luciferase reporter gene assay results were expressed as the mean ± SD of determinations made in three different experiments. Probability (*p*) values were calculated by *t*-test. A *p* value of less than 0.05 was considered statistically significant.

## Supporting information

S1 Fig Toscana virus infection leads to RIG-I production. Endogenous RIG-I protein level was observed by western blotting. Lenti-X 293T cells were stimulated with polyI:C transfection for 18h, mock-infected or infected with TOSV (MOI = 1). Where indicated, stimulated or infected cells were either transfected with empty plasmid or plasmid expressing wt-NSs. Cell lysates were prepared at indicated times post-infections and 50 μg of total proteins were resolved by SDS-PAGE and assessed for RIG-I expression by specific antibody. TOSV NSs expression, along with nucleoprotein N, was determined on the same lysates to confirm viral infection and replication. Band intensity was determined by densitometric analysis performed on at least three independent experiments. Results are given in Supplement data 1.
(TIF)

S2 Fig TOSV NSs amino-acid sequence. Full-length NSs amino-acidic sequence showing the amino-terminal (NSsΔN) and the carboxy-terminal (NSsΔC) deleted mutants of the protein. The functional active Cysteine residue at position 27 is shown in bold.
(TIF)

S3 Fig Toscana virus NSs protein retains E3 ubiquitin ligase activity on RIG-I. Recombinant NSs and RIG-I proteins were used in combination with E1 ubiquitin activating enzyme, UbcH5b/c E2 ubiquitin conjugating enzyme and wt-rNSs, as source of E3 ubiquitin ligase, in the ubiquitination assay *in vitro*. Target protein for ubiquitination was represented by the full-length human rRIG-I. Negative controls, including the recombinant TOSV viral nucleoprotein (rN) or the omission of ATP energy source, were included. The presence of poly-ubiquitinated rRIG-I was revealed with anti-ubiquitin or anti-RIG-I antibodies represented by an increase of the specific molecular weight.
(TIF)

S4 Fig Sequence alignment of TOSV and SFNV NSs proteins. Comparison of the amino acid sequences of Toscana virus (TOSV; strain 1812, GenBank Accession N° ABY19522.1) and Sandfly Fever Naples virus (SFNV; strain Sabin, GenBank Accession N° EF201829) NSs showing the homology (54%) between the two related viral proteins and the lack of TOSV C-terminal domain in SFNV NSs.
(TIF)

S5 Fig Tracking of RIG-I CARDs ubiquitination by mass spectrometry. Confirmatory results of RIG-I CARDs ubiquitination by TOSV NSs were obtained by the mass spectrometry analysis. The ≥ 40 KDa fraction of the biochemical reaction products revealed the presence of ubiquitinated RIG-I peptides only in samples supplemented with wt-rNSs or cSFNV NSs. Moreover, this approach allowed the identification of RIG-I CARDs lysine residues 115 and 172 as target for ubiquitination by the NSs.
(TIF)

S6 Fig Mass spectrum of Toscana virus NSs protein. A cell line derived from Lenti-X 293T cells stably expressing Toscana virus NSs protein was used for purification under denaturing conditions of the viral protein. The enriched substrate protein was subjected to mass spectrum showing the identification of TOSV NSs peptide containing the ubiquitinated Cysteine residue at position 27 (Cys_27_).
(TIF)

S7 Fig C_27_G-NSs mutant is unable to mediate RIG-I rCARDs ubiquitination. The key role of C_27_ in the N-terminus of TOSV NSs was further investigated by *in vitro* ubiquitination of RIG-I rCARDs. Higher molecular weight bands corresponding to rCARDs ubiquitinated forms were detected by both anti-RIG-I and anti-Ub antibodies only when the wt-NSs was used in the biochemical reaction. On the contrary, C_27_G-NSs mutant was unable to mediate RIG-I rCARDs ubiquitination, confirming a direct involvement of the C_27_ in the ubiquitination process. Asterisk in the sample containing wt-NSs indicates ubiquitinated rRIG-I CARDs, as reported by mass spectrometry (S5 Fig). On the contrary, the corresponding immune-reactive bands evidenced in other samples were identified as the E2-Ub intermediate.
(TIF)

S1 Dataset Evaluation of TOSV effects on endogenous RIG-I expression. Immunoblotting for detection of endogenous RIG-I expression in TOSV infected, poly(I:C) and NSs transfected Lenti-X 293T cells were subjected to densitometric analysis. Raw dataset of RIG-I, TOSV NSs and actin band intensity was reported from three independent experiments. After normalization with respect to relative actin values, a comparison was performed and protein expression levels ± standard deviation (SD) were calculated as fold induction. A *p* value of less than 0.05 was considered statistically significant.
(XLS)

S2 Dataset Ubiquitination activity of wt NSs and NSs deleted variants. Lenti-X 293T cells were transfected with RIG-I or p53 expressing plasmids, alone or in combination to wt-NSs or its deleted mutants. Quantification of RIG-I or p53 expression levels was performed by densitometric analysis on immunoblotting and raw dataset of RIG-I, p53, NSs and actin band intensity were reported from three independent experiments. After normalization with respect to relative actin values, a comparison was performed and protein expression levels ± standard deviation (SD) were calculated as fold induction. Moreover, specificity of wt-NSs was assessed by immunofluorescence in p53 plasmid co-transfected cells. Both p53 or NSs positive cells were counted and percentage was calculated ± standard deviation (SD). The influence of NSs deleted mutants on RIG-I-mediated IFN-β promoter activation was assessed by Luciferase reporter gene assay. Fold induction of IFN-β promoter activation was reported from three independent experiments ± standard deviation (SD). A *p* value of less than 0.05 was considered statistically significant.
(XLS)

S3 Dataset C-terminal domain of TOSV NSs is associated to its ubiquitination function. Quantification of RIG-I cellular accumulation was performed by densitometric analysis on immunoblotting from Fig 2. Raw dataset of RIG-I, TOSV or SFNV NSs, chimeric cSFNV NSs and actin band intensity was listed from three independent experiments. After normalization with respect to relative actin values, fold induction/decrease in protein expression levels ± standard deviation (SD) was calculated. Immunofluorescence data referring to RIG-I or NSs positive cells were given and final results were expressed as percentage of positive cells with respect to the total number of spotted cell. A more accurate analysis was performed by Luciferase reporter gene assay by which the effects of different NSs variants on RIG-I-mediated IFN-β promoter activation was evaluated. Fold induction was calculated for each sample with respect to the basal empty plasmid transfected sample, after normalization of the signal with the pSV40-RenLuc internal control. The mean values of at least three sets of experiments ± SD were presented. For all the experimental procedures a *p* value of less than 0.05 was considered statistically significant.
(XLS)

S4 Dataset C_27_ residue at the TOSV NSs N-terminal domain is critical for its E3 ubiquitin ligase activity. RIG-I expression levels were quantified by densitometric analysis on immunoblotting performed on cell lysates of RIG-I and NSs, wild-type or cystein mutant, co-transfected cells. Raw dataset represented the band intensity for RIG-I, wt-NSs, NSsC_27_G and actin. Fold induction/decrease in protein expression levels was calculated after actin normalization. Immunofluorescence results performed for RIG-I or NSs immune-staining were given. Positive cells for both RIG-I or NSs were counted; results were expressed as percentage of positive cells with respect to the total number of tested cells. Reporter gene assay was used to determine the effects of cysteine mutated NSs on RIG-I-mediated IFN-β promoter activation. Luciferase reporter gene assay was performed and fold induction in IFN-β promoter activation was calculated for each sample after normalization of the signal with the pSV40-RenLuc internal control. For all the experimental procedures, results were collected from three independent experiments and expressed as mean values ± standard deviations (SD). A *p* value of less than 0.05 was considered statistically significant.
(XLS)
