# Supplementary figures and images for: Toscana virus non-structural protein NSs acts as E3 ubiquitin ligase promoting RIG-I degradation
Source: PLoS Pathog. 2019 Dec 9;15(12):e1008186. doi: 10.1371/journal.ppat.1008186 (PMC6901176; doi:10.1371/journal.ppat.1008186)

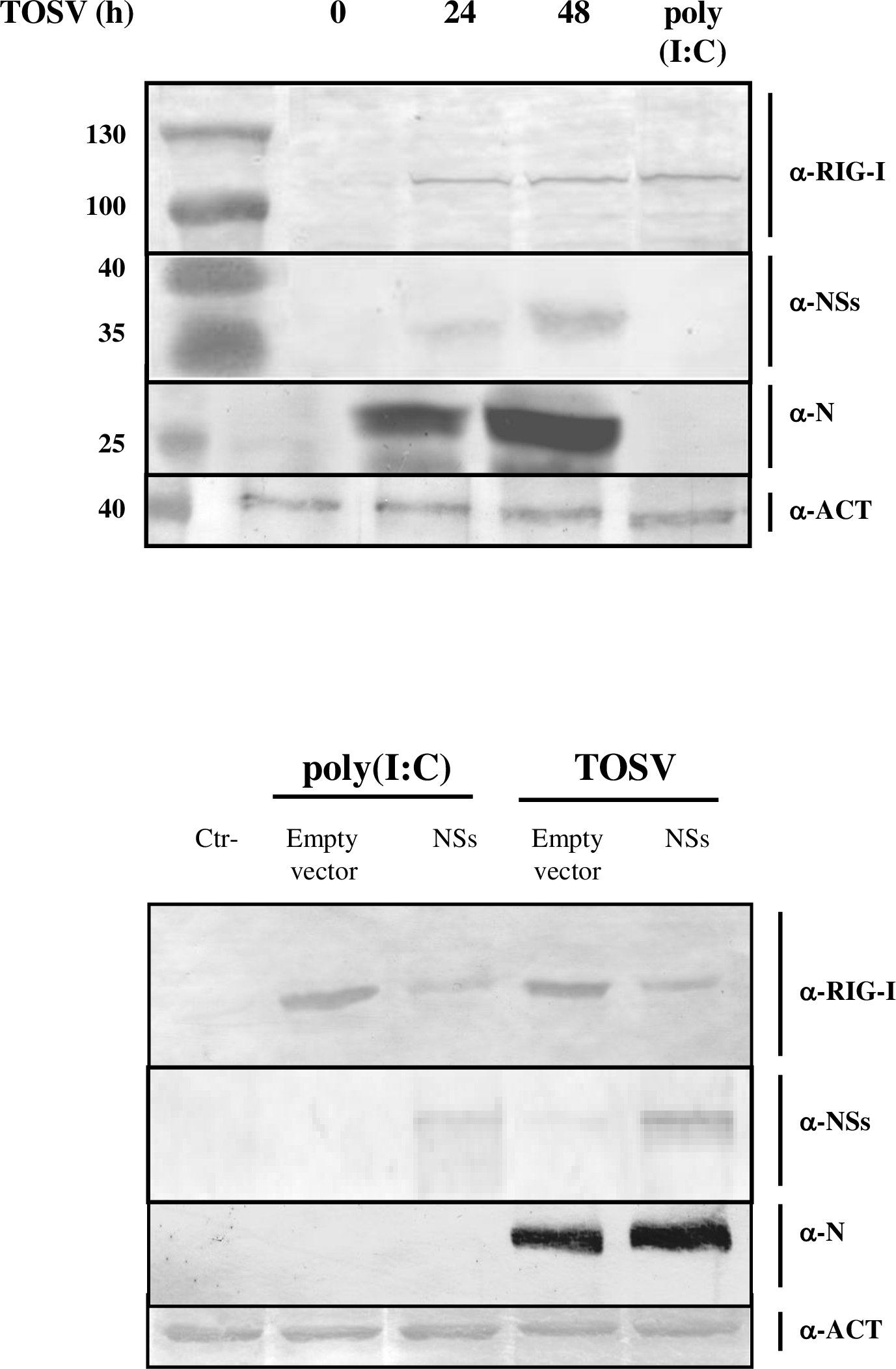

Supplement: S1 Fig — Endogenous RIG-I protein level was observed by western blotting. Lenti-X 293T cells were stimulated with polyI:C transfection for 18h, mock-infected or infected with TOSV (MOI = 1). Where indicated, stimulated or infected cells were either transfected with empty plasmid or plasmid expressing wt-NSs. Cell lysates were prepared at indicated times post-infections and 50 μg of total proteins were resolved by SDS-PAGE and assessed for RIG-I expression by specific antibody. TOSV NSs expression, along with nucleoprotein N, was determined on the same lysates to confirm viral infection and replication. Band intensity was determined by densitometric analysis performed on at least three independent experiments. Results are given in Supplement data 1. (TIF) [file ppat.1008186.s001.tif]

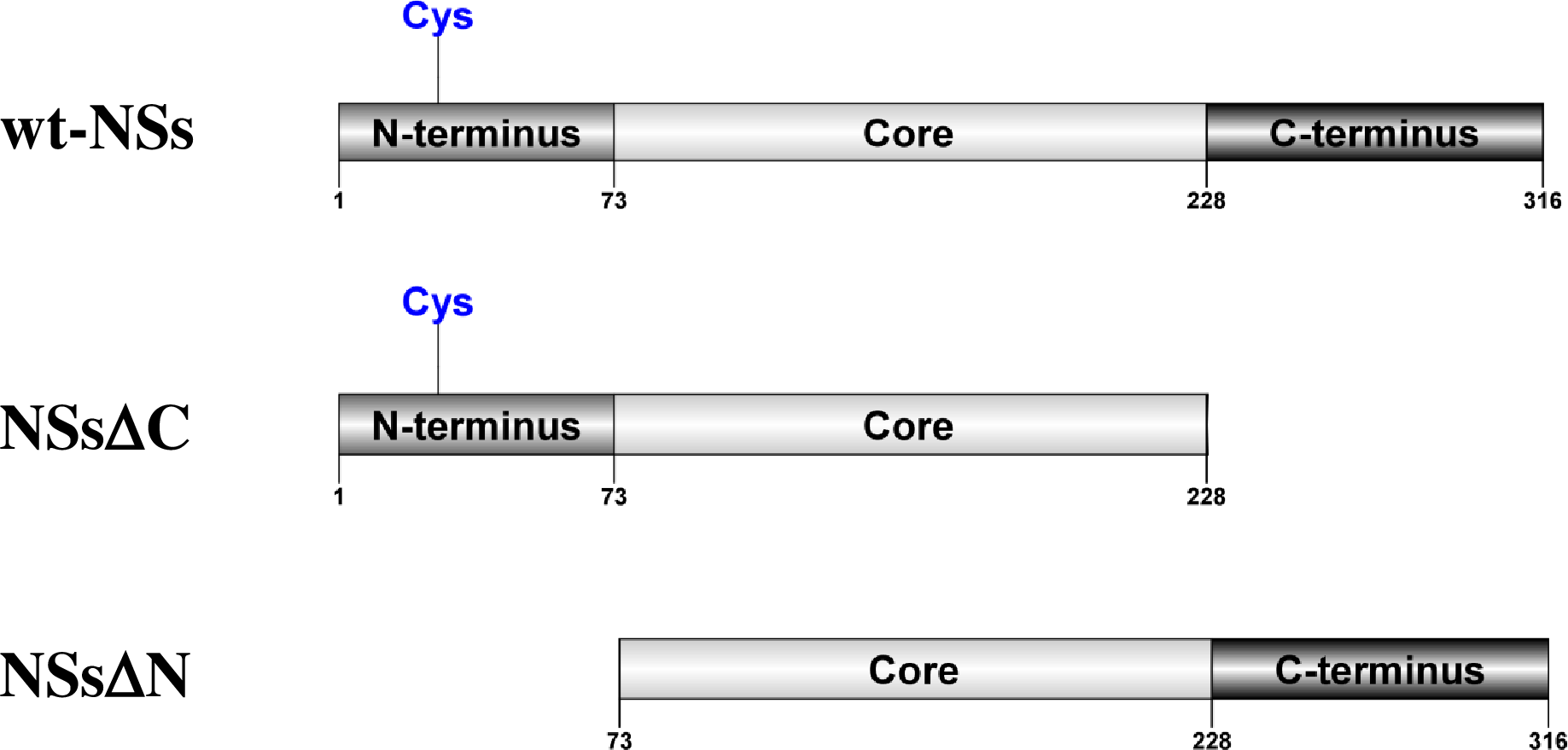

Supplement: S2 Fig — Full-length NSs amino-acidic sequence showing the amino-terminal (NSsΔN) and the carboxy-terminal (NSsΔC) deleted mutants of the protein. The functional active Cysteine residue at position 27 is shown in bold. (TIF) [file ppat.1008186.s002.tif]

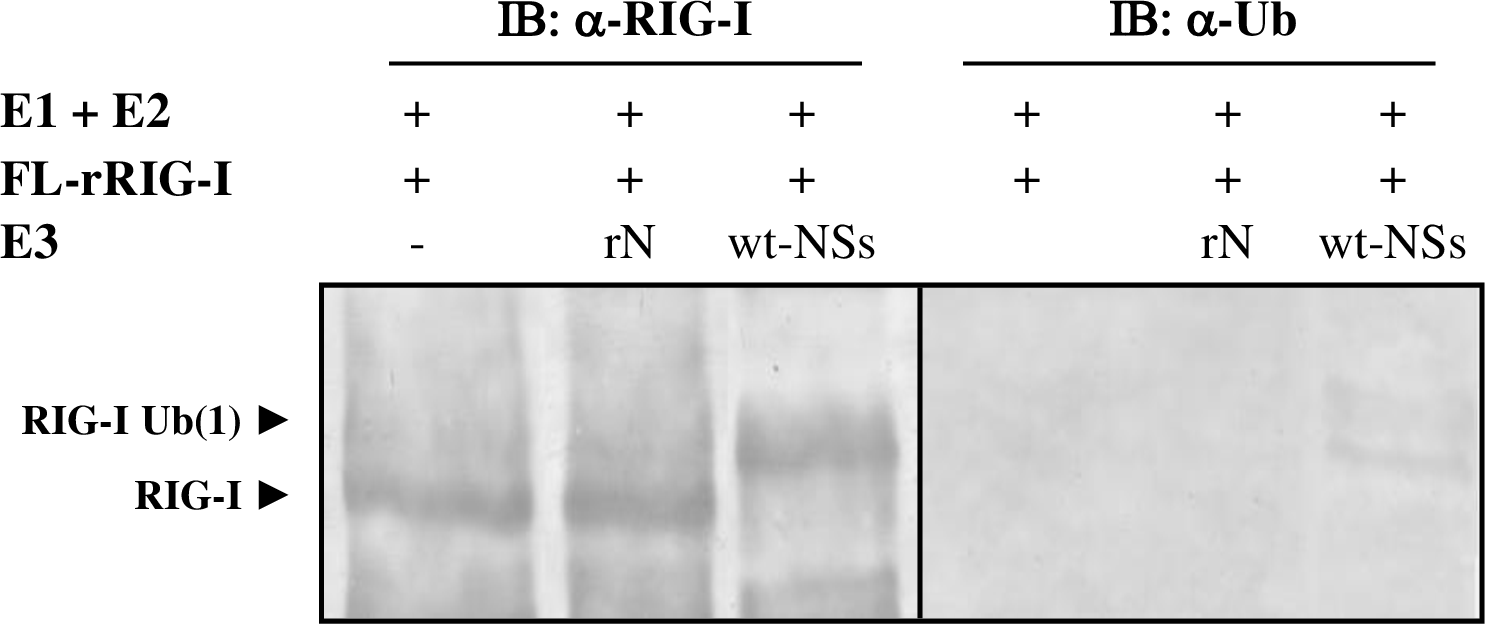

Supplement: S3 Fig — Recombinant NSs and RIG-I proteins were used in combination with E1 ubiquitin activating enzyme, UbcH5b/c E2 ubiquitin conjugating enzyme and wt-rNSs, as source of E3 ubiquitin ligase, in the ubiquitination assay in vitro. Target protein for ubiquitination was represented by the full-length human rRIG-I. Negative controls, including the recombinant TOSV viral nucleoprotein (rN) or the omission of ATP energy source, were included. The presence of poly-ubiquitinated rRIG-I was revealed with anti-ubiquitin or anti-RIG-I antibodies represented by an increase of the specific molecular weight. (TIF) [file ppat.1008186.s003.tif]

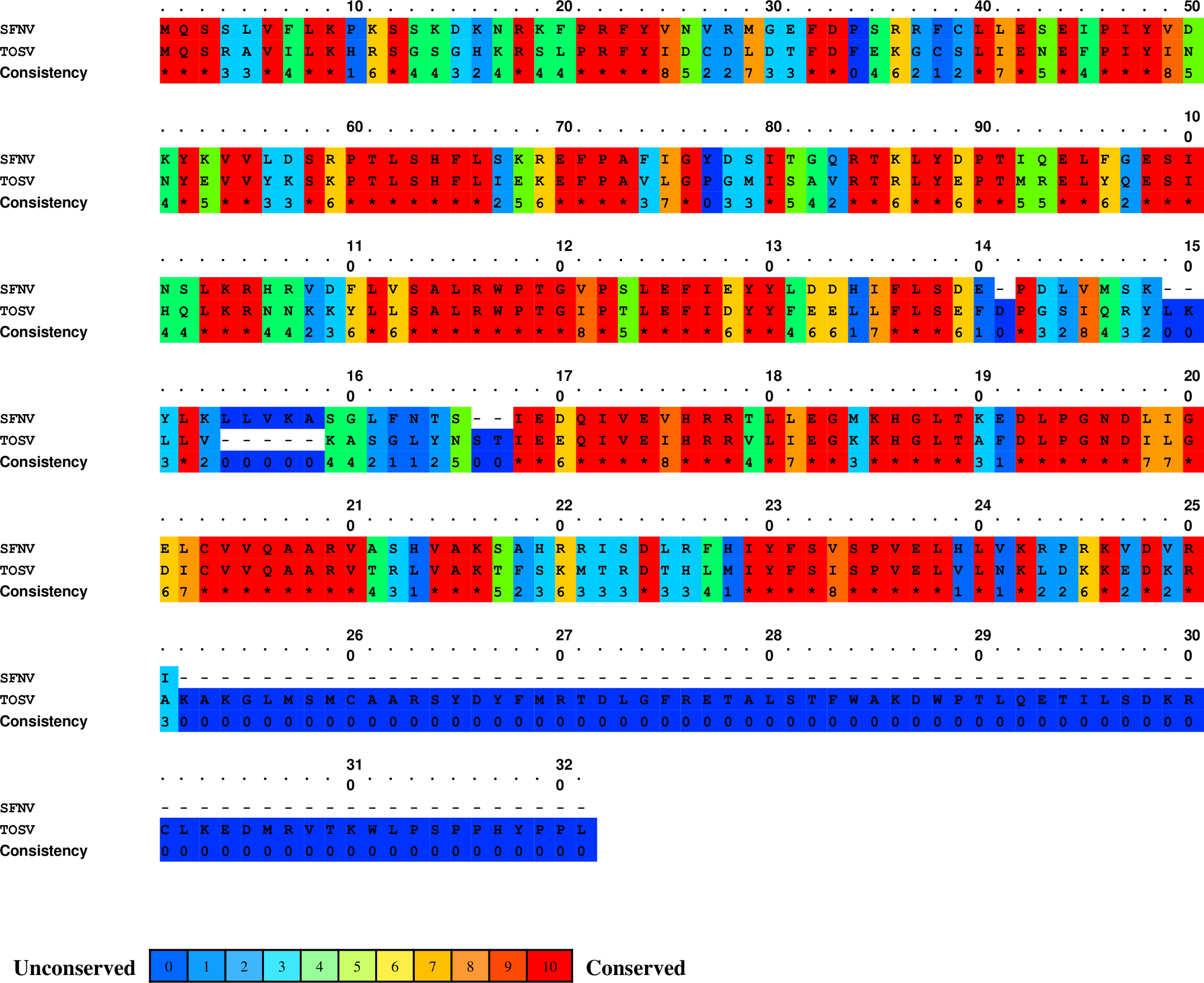

Supplement: S4 Fig — Comparison of the amino acid sequences of Toscana virus (TOSV; strain 1812, GenBank Accession N° ABY19522.1) and Sandfly Fever Naples virus (SFNV; strain Sabin, GenBank Accession N° EF201829) NSs showing the homology (54%) between the two related viral proteins and the lack of TOSV C-terminal domain in SFNV NSs. (TIF) [file ppat.1008186.s004.tif]

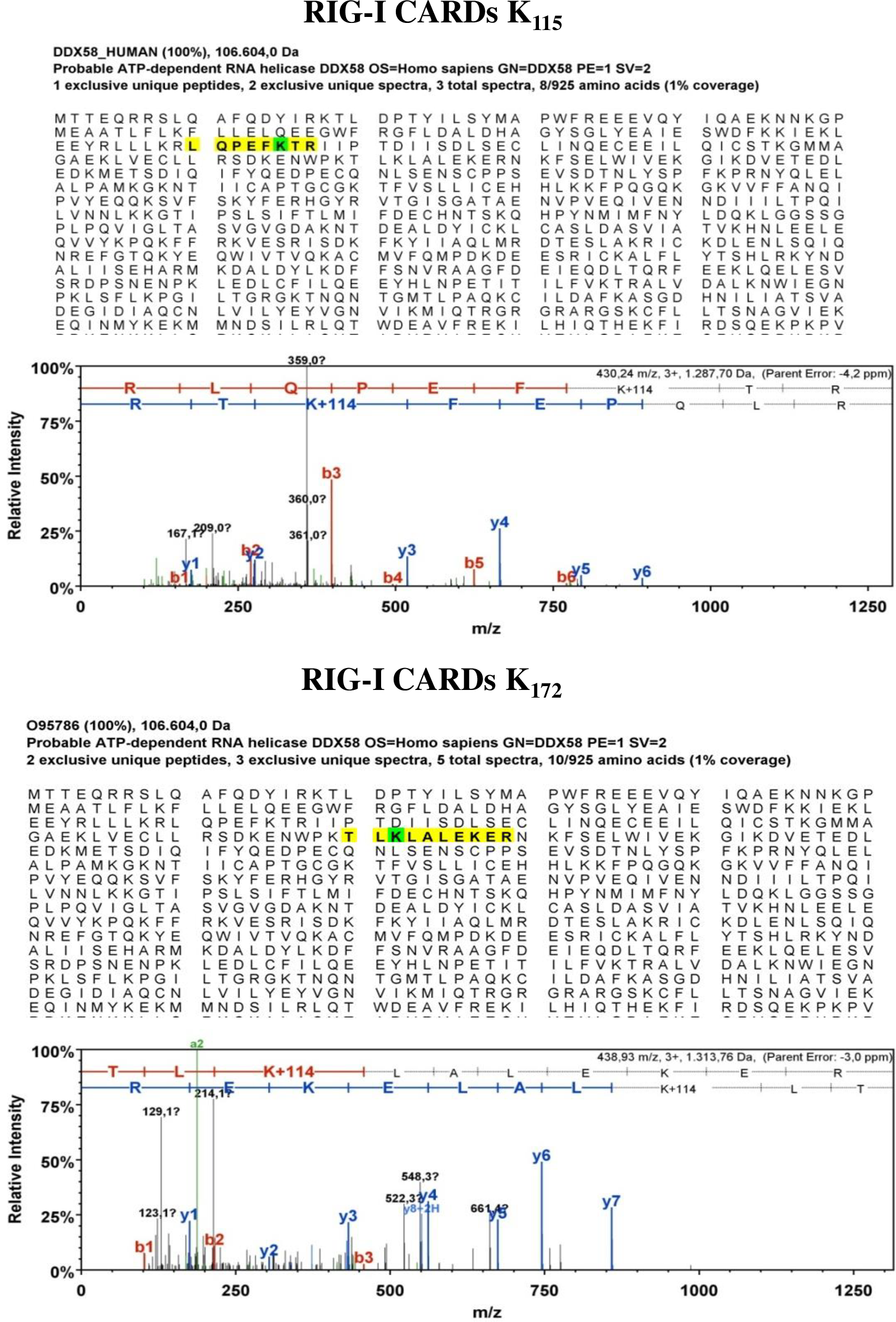

Supplement: S5 Fig — Confirmatory results of RIG-I CARDs ubiquitination by TOSV NSs were obtained by the mass spectrometry analysis. The ≥ 40 KDa fraction of the biochemical reaction products revealed the presence of ubiquitinated RIG-I peptides only in samples supplemented with wt-rNSs or cSFNV NSs. Moreover, this approach allowed the identification of RIG-I CARDs lysine residues 115 and 172 as target for ubiquitination by the NSs. (TIF) [file ppat.1008186.s005.tif]

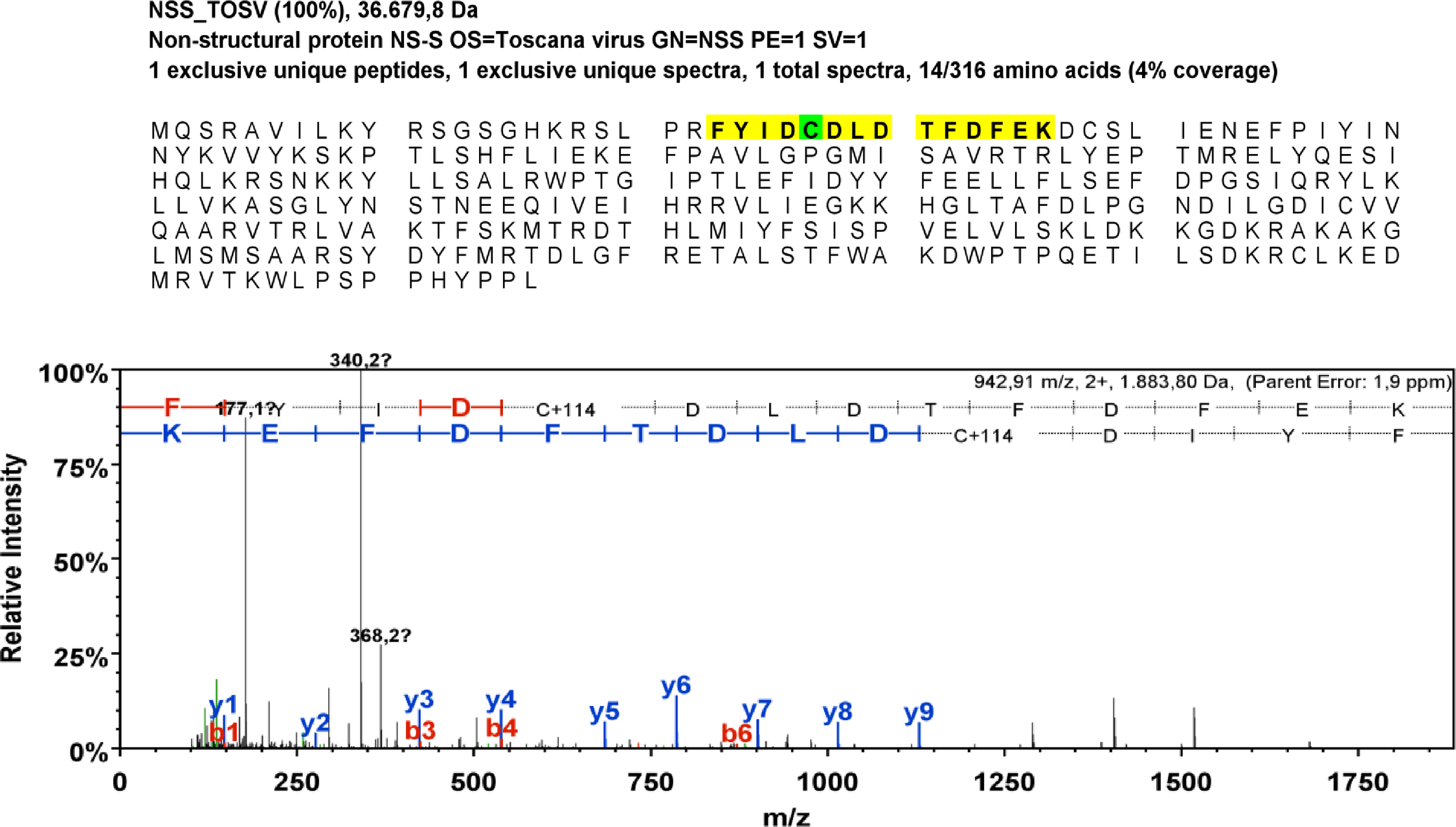

Supplement: S6 Fig — A cell line derived from Lenti-X 293T cells stably expressing Toscana virus NSs protein was used for purification under denaturing conditions of the viral protein. The enriched substrate protein was subjected to mass spectrum showing the identification of TOSV NSs peptide containing the ubiquitinated Cysteine residue at position 27 (Cys27). (TIF) [file ppat.1008186.s006.tif]

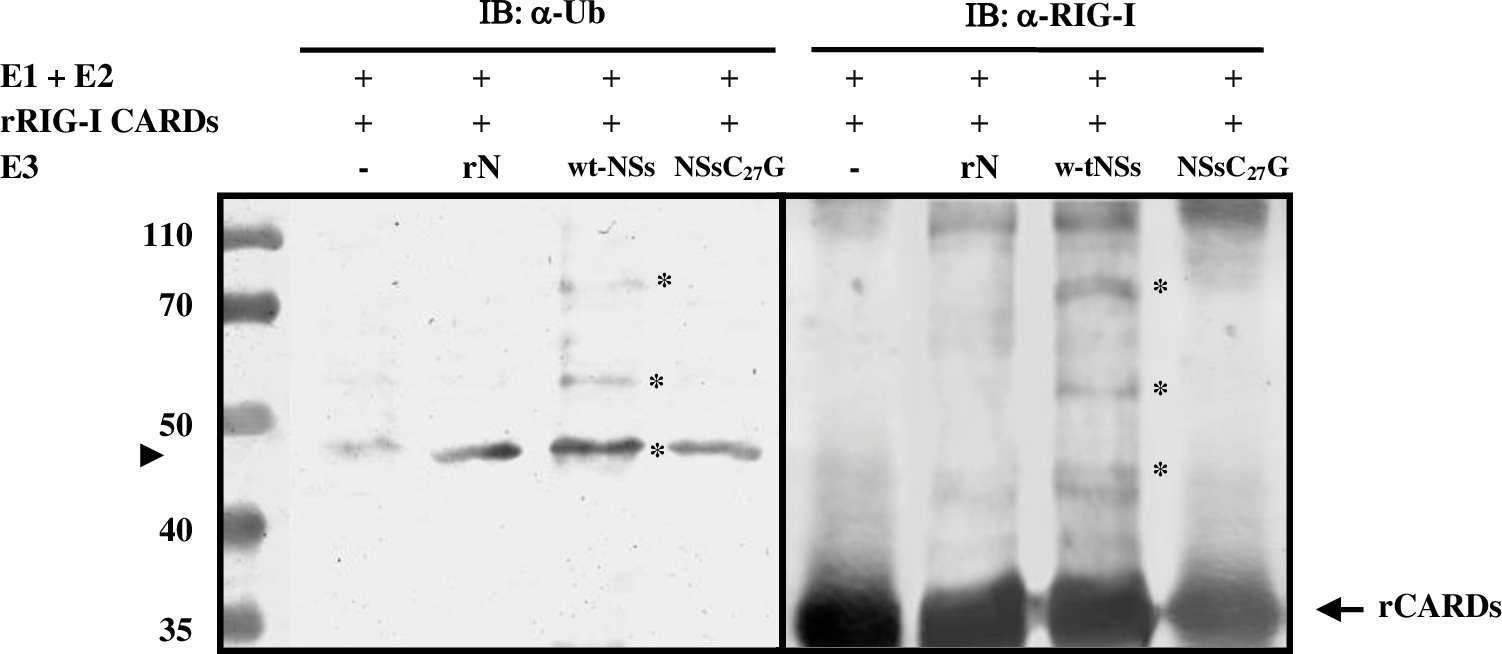

Supplement: S7 Fig — The key role of C27 in the N-terminus of TOSV NSs was further investigated by in vitro ubiquitination of RIG-I rCARDs. Higher molecular weight bands corresponding to rCARDs ubiquitinated forms were detected by both anti-RIG-I and anti-Ub antibodies only when the wt-NSs was used in the biochemical reaction. On the contrary, C27G-NSs mutant was unable to mediate RIG-I rCARDs ubiquitination, confirming a direct involvement of the C27 in the ubiquitination process. Asterisk in the sample containing wt-NSs indicates ubiquitinated rRIG-I CARDs, as reported by mass spectrometry (S5 Fig). On the contrary, the corresponding immune-reactive bands evidenced in other samples were identified as the E2-Ub intermediate. (TIF) [file ppat.1008186.s007.tif]
